# Recompensation of Decompensated Hepatitis B Cirrhosis: Current Status and Challenges

**DOI:** 10.1155/2020/9609731

**Published:** 2020-09-21

**Authors:** Hong Zhao, Qi Wang, Changling Luo, Ligai Liu, Wen Xie

**Affiliations:** Liver Diseases Center, Beijing Ditan Hospital, Capital Medical University, 8 East Jingshun Road, Chaoyang District, Beijing, China

## Abstract

Liver-function decompensation or hepatocellular carcinoma (HCC) gradually appears after chronic hepatitis B progresses to cirrhosis. Effective antiviral treatment can significantly improve the long-term prognosis of decompensated patients, and some patients present recompensation of decompensated hepatitis B cirrhosis. At present, there are limited research data on the recompensation of decompensated hepatitis B cirrhosis. There is still controversy regarding the evaluation time, evaluation indicators, influencing factors, and long-term prognosis of recompensation.

## 1. Introduction

The World Health Organization reported that there are an estimated 240 million people globally with chronic hepatitis B virus (HBV) infection. Approximately 2% to 4% of patients develop compensated cirrhosis each year without effective treatment. Each year, approximately 1.5% to 4% of patients with cirrhosis further develop decompensated cirrhosis (with symptoms such as ascites, hepatic encephalopathy, and gastrointestinal-varix bleeding), leading to repeated hospitalization, severe reduction in quality of life, and even death. As a result of cirrhosis, hepatocellular carcinoma (HCC) occurs in approximately 3% to 6% of patients [1, 2]. Patients with decompensated cirrhosis have a higher rate of liver transplantation, mortality, and HCC, and a worse prognosis [3, 4]. Effective antiviral therapy can inhibit the hepatitis B virus (HBV) replication, improve liver function in patients with decompensated cirrhosis [5], and recompensate liver function in some patients [6], thereby improving their quality of life, prolonging survival time, and reducing the burden of HBV-related diseases [7–9]. In this paper, the current research status, problems, and challenges of the recompensation of decompensated hepatitis B cirrhosis are reviewed.

## 2. Definition of the Recompensation of Decompensated Hepatitis B Cirrhosis

Liver cirrhosis is usually divided into compensated and decompensated phases on the basis of whether patients with hepatitis B experienced severe complications such as ascites, gastrointestinal-varix bleeding, and hepatic encephalopathy [10]. Untreated patients with decompensated hepatitis B cirrhosis were previously reported to have poor prognosis, with a five-year survival rate of only 14% to 35% [11, 12]. The long-term prognosis of decompensated hepatitis B cirrhosis can be improved with increased levels of symptomatic and supportive therapies, and the active use of antiviral drugs [7].

Clinically, some patients with decompensated hepatitis B cirrhosis demonstrate significant improvements in liver function and a reduction in portal-hypertension-related complications through effective antiviral therapies. Patients are stable for a long time. They do not develop syndromes similar to compensated cirrhosis, such as ascites, gastrointestinal-varix bleeding, and hepatic encephalopathy, which are considered to be “recompensation” for the development of decompensated hepatitis B cirrhosis.

According to the Chinese Society of Hepatology Guidelines for the Diagnosis and Treatment of Cirrhosis, cirrhosis patients may no longer have decompensated cirrhosis events (such as ascites, gastrointestinal bleeding, and hepatic encephalopathy) for a long period of time (at least one year) due to effective etiology control and the effective treatment or prevention of complications. Moreover, there may still be clinical and laboratory characteristics of compensated cirrhosis. This situation can be considered recompensation of decompensated hepatitis B cirrhosis.

Recompensation of decompensated hepatitis B cirrhosis is a state in which, after a period of active treatment, the liver's reserved function can meet the patient's daily activities, and no complications related to cirrhosis decompensation occur. In this state, the liver disease of the patient has no obvious progress or improvement, and it is not clear whether it can be maintained for a long period of time. From a pathological point of view, there is no sufficient clinical evidence to support that liver fibrosis in patients with decompensated hepatitis B cirrhosis can be reversed.

## 3. Current Status of Recompensation of Hepatitis B Cirrhosis Decompensation

At present, there are few studies on the recompensation of decompensated hepatitis B cirrhosis. After combining the literature on the clinical efficacy of antiviral therapies for decompensated hepatitis B cirrhosis, the research status of the recompensation of decompensated hepatitis B cirrhosis was summarized, as shown in Table 1. We list several studies related to oral antiviral therapy in HBV-related decompensated cirrhosis. In these studies, the number of cases was more than 50, and the follow-up time was more than 1 year. These studies included different patient populations with different severity in terms of Child–Turcotte–Pugh (CTP) or model for end-stage liver disease (MELD) scores, while the trials had different aims/designs. Most of these studies lacked a specific description of the occurrence of complications in decompensated liver cirrhosis. The occurrence of complications is very important for evaluating the therapeutic effect of decompensated hepatitis B liver cirrhosis.

Shim et al. [13] enrolled 70 HBV-infected patients with decompensated cirrhosis who were primarily treated with 0.5 mg of entecavir (ETV) daily, and they evaluated the clinical outcomes using intention-to-treat analyses. Cumulative transplantation-free survival was 87.1% at one year. ETV treatment for 12 months resulted in improved Child–Turcotte–Pugh (CTP) and model for end-stage liver disease (MELD) scores. In total, 66% (36/55) of patients achieved CTP Class A, and 49% (27/55) showed an improvement in CTP score of two points after 12 months of ETV.

A randomized, open-label comparative study of ETV versus adefovir therapy was performed by Liaw et al. [14], involving subjects with chronic hepatitis B who had hepatic decompensation (CTP score ≥ 7). Adult subjects were randomized and treated (*n* = 191) with 1.0 mg of ETV or 10 mg of adefovir daily for up to 96 weeks from the date of the last subject randomization. Approximately two-thirds of the subjects in both groups showed improvement and stabilization in CTP status. MELD score changes at week 48 were −2.6 for ETV and −1.7 for adefovir. Among those with baseline hepatic encephalopathy, clinical improvement was observed in 17/22 (77.3%) ETV-treated and 10/23 (43.5%) adefovir dipivoxil- (ADV-) treated patients. Similarly, in patients with baseline ascites, reversal was seen in 26/63 (41.3%) ETV-treated and 23/61 (37.7%) ADV-treated patients. Cumulative death rates were 23% for ETV and 33% for adefovir. Week 24 mortality rates were 12% for both groups.

Singal and Fontana [15] performed a meta-analysis of one-year efficacy and safety outcomes in 22 studies conducted between 1995 and 2010 on oral nucleotide analogs in patients with decompensated HBV cirrhosis. Pooled one-year data showed a favorable benefit of ETV (lamivudine; LAM) vs. untreated controls. CTP score was improved by ≥2 (odds ratio (OR): 117 (15, 921), *p* ≤ 0.0001). Transplant-free survival was also improved (OR: 3.2 (1.2, 9), *p* = 0.022). Overall, one-year transplant-free survival rates ranged from 78% with LAM to 95% and 94% with tenofovir (TDF) and telbivudine (LdT), respectively. All oral antiviral agents were associated with improved virological, biochemical, and clinical parameters at one year. However, the efficacy of ETV and LdT was compromised by drug resistance. In addition, adefovir had low potency and a slower onset of action.

Srivastava et al. [16] evaluated the usefulness of various prognostic indicators in predicting the 24-month survival of patients with HBV-related decompensated cirrhosis after tenofovir (TDF) therapy, as well as the posttreatment outcome. The 24-month survival and mortality of 96 HBV-related decompensated patients were studied after TDF therapy. Overall survival was 0.947 at 12 months and 0.833 at 24 months. Multivariate analysis showed that an MELD score > 20 was the most robust predictor of mortality. Reversal of decompensation was observed in 48.6% of cases at the end of 24 months (i.e., without ascites or any other feature of liver failure). Posttreatment response with 24 months of TDF therapy was significantly improved in terms of hepatic function, with reversed decompensation. It showed incredible efficacy in the improvement of hepatic functional status with reduced viremia in a great majority of decompensated cirrhosis subjects who had high MELD and HBV DNA levels.

Yue-Meng et al. [17] retrospectively evaluated 130 treatment-naïve patients with HBV-related decompensated cirrhosis who had started treatment with telbivudine (LdT; *n* = 31), lamivudine (LAM; *n* = 45), or entecavir (*n* = 54). After 24 months of treatment, CTP and MELD scores were significantly decreased in all groups from 12 months onward in comparison to the baseline. Cumulative survival rates at 24 months were 80%, 93.3%, and 86.8% in the LdT, LAM, and ETV groups, respectively (*p* = 0.222, log-rank test). During the study, 16 patients died of the following causes: variceal bleeding (*n* = 6), liver failure (*n* = 6), pneumonia (*n* = 1), spontaneous bacterial peritonitis (*n* = 1), and HCC metastasis to the lungs (*n* = 2). Nineteen patients developed HCC. The cumulative rates of HCC development at 24 months were 15.0%, 14.0%, and 13.5% in the LdT, LAM, and ETV groups, respectively.

Jang et al. [18] performed a 10-year observation analysis using data from the Epidemiology and Natural History of Liver Cirrhosis study of patients with decompensated liver cirrhosis in Korea. Of the entire cohort (1595 patients enrolled at the onset of decompensation since 2005), their analysis comprised 295 patients. In total, 60.1% of patients survived for five years and 45.7% survived for 10 years without liver transplantation. Maintained virologic response (MVR, defined as persistent undetectable HBV DNA during therapy) was observed in 116 patients (39.3%); these patients had significantly longer transplant-free survival than those of patients without an MVR. Baseline MELD score > 20 and multiple complications were associated with short-term mortality. MVR was the factor that had the strongest association with long-term transplant-free survival. Patients with an MVR had significant improvement in hepatic function over time. However, no significant reduction in the risk of HCC or HCC-related mortality was observed in these patients.

As can be seen from the above, currently published studies on the efficacy of antiviral therapy for decompensated hepatitis B cirrhosis mainly focused on comparisons of the efficacy of different antiviral agents during an observation time of one to two years. The only study with a 10-year follow-up cohort was the Korean study. Results in this study suggested that a virologic response was achieved in most patients after active antiviral therapy. Treated patients demonstrated an improvement in liver-function-related measures [13, 14, 17, 19–23]. The long-term efficacy of patients was generally assessed on the basis of a reduction in MELD and CTP scores [17–19] or the incidence of HCC, liver transplantation, and liver-disease-related death. Study results showed that MELD and CTP scores in patients with decompensated hepatitis B cirrhosis were decreased after effective antiviral therapy, suggesting that some patients may be recompensated for cirrhosis. However, only a few studies mentioned the complications related to decompensated cirrhosis. Therefore, at present, there are not many data on the recompensation of decompensated hepatitis B cirrhosis, and whether it can reduce the occurrence of HCC is controversial. The long-term prognosis of these patients is not clear.

## 4. Issues and Challenges

### 4.1. Complexity in Mechanisms of Recompensation of Decompensated Hepatitis B Cirrhosis

Hepatic-function decline and portal hypertension are the most important pathophysiological changes observed in decompensated cirrhosis. Several studies showed that effective antiviral therapies can improve liver function in patients with decompensated hepatitis B cirrhosis and help to recompensate cirrhosis. Severe portal hypertension can cause uncontrolled or recurring complications of decompensated liver cirrhosis, causing a significant reduction in survival rate without liver transplantation. The hepatic venous pressure gradient (HVPG) indirectly reflects portal-vein resistance. Studies showed that, in patients with portal hypertension, HVPG is reduced by at least 20% or to below 12 mmHg from the baseline using medication/nondrug treatment, which significantly reduces the risk of bleeding and the incidence of decompensation or progressive decompensation; risk of death is also significantly reduced. Effective antiviral therapy can reduce portal pressure and the risk of bleeding in some patients [24].

The pathogenesis of complications of decompensated hepatitis B cirrhosis is very complicated. Under portal hypertension, the formation of portal collateral circulation and the occurrence of a portosystemic shunt are promoted. The formation of portal hypertension increases the risk of ascites and esophagogastric varices. The formation of a portal shunt increases the risk of hepatic encephalopathy. Research by Nagaoki et al. [25] found that, even in patients with hepatitis B cirrhosis who responded well to antiviral therapy, baseline portal-vein collateral circulation and the extrahepatic portal shunt still had a higher incidence of esophagogastric-varix exacerbation and a risk of portal-venous systemic shunt-associated hepatic encephalopathy. Patients with liver cirrhosis have decreased resistance and are more easily infected. Studies showed that infection increases the mortality of patients with cirrhosis fourfold, resulting in patient death within one month of infection in 30% of cases [26]. Hepatorenal syndrome is a serious complication of liver cirrhosis. Patients with liver cirrhosis show a sevenfold increase in mortality, with 50% of patients dying within one month [27]. Therefore, patients with repeated complications often have poor prognosis.

In fact, the clinical manifestations of patients with decompensated hepatitis B cirrhosis are different. Some patients may present with massive ascites, while others may present with variceal bleeding or recurrent hepatic encephalopathy, and a few may present with hepatorenal syndrome and hepatopulmonary syndrome. The nutritional problems of patients with chronic liver disease are also receiving increased attention [28]. Sarcopenia may be considered one of the most common and significant complications of liver cirrhosis, and it is associated with adverse outcomes and increased morbidity and mortality [29].

Comprehensive treatment of complications can also affect the incidence of recompensation, such as the use of diuretics, portal-vein pressure-lowering drugs, endoscopic treatment of esophagogastric varices, shunt or devascularization of the portal-vein system, splenectomy, and nutritional support. These treatments affect the occurrence and duration of complications and change the long-term prognosis of patients with cirrhosis. However, it is unclear whether different types of complications need to be separately investigated.

### 4.2. Lack of Objective Evaluation Indicators for Recompensation of Decompensated Hepatitis B Cirrhosis

Previous studies showed that partially decompensated patients with hepatitis B cirrhosis can achieve cirrhosis recompensation through effective antiviral treatment. However, not all patients can achieve cirrhosis recompensation by inhibiting HBV replication. Some patients still have bad prognosis [30, 31]. Jang et al. [18] reported that, among 295 patients with decompensated hepatitis B cirrhosis who had started antiviral therapy at the time of first decompensation, 20 patients (6.8%) died of cirrhosis-related complications within six months of antiviral therapy. Fontana et al. [32] prospectively enrolled 154 patients with decompensated hepatitis B cirrhosis. After treatment with LAM, patients had a median follow-up of 16 months (0.5–37 months). Most deaths (78%) occurred in the first six months after initiation of antiviral therapy, with cirrhosis-related complications as the primary cause of death. The three-year survival rate is 88% in patients treated with antiviral therapy for more than six months. Thus, patients with severely decompensated cirrhosis might not be recompensated, and they may even die before a virologic response. It is essential to promptly identify high-risk patients and implement effective treatment strategies.

It is reported in the literature that antiviral therapy for one year can reduce the score of patients with decompensated hepatitis B cirrhosis who have a baseline CTP score of ≥7 points. The treatment can also decrease the score by ≥2 points or by 49% to 72% [15]. A MELD score of >20 is considered to be the most effective predictor of death in patients with decompensated hepatitis B cirrhosis treated with TDF (the two-year mortality rate of patients with MELD score > 20 and <20 points is 60% and 1.4%, respectively) [16]. Similarly, the baseline CTP score and the MELD score after three months of antiviral treatment can predict a patient's six-month mortality rate. In liver transplantation, although the CTP score at three months is not statistically different between the death and survival groups, the survival group had a higher score than that of the death group. CTP score decreases in the first six months after treatment, but the decrease is not significant afterward [31]. Other prediction methods with important potential include the end-stage liver-disease-model dynamic score (*Δ*MELD) and MELD combined with serum sodium, APRI, and FIB-4.

Therefore, a comprehensive evaluation index of liver function may be helpful for the early identification of patients with “recompensation advantage.” The CTP score integrates the two aspects of liver function and complications, and it can be dynamically monitored. We speculated that a dynamic change in CTP score may be a good early evaluation indicator, but it cannot reflect the dynamic changes in complications such as gastrointestinal-varix bleeding, hepatorenal syndrome, hepatopulmonary syndrome, and sarcopenia.

### 4.3. Lack of Liver-Pathology Research to Support Recompensation of Decompensated Hepatitis B Cirrhosis

Liver histology remains the gold standard for the diagnosis of cirrhosis. Histological evaluation of liver cirrhosis can be divided into active and quiescent periods. In the Laennec cirrhosis scoring system that is commonly recommended, the pathological diagnosis of liver cirrhosis can be further divided into Laennec 4A, 4B, and 4C substages according to the width of fibrous septa and the size of sclerosing nodules [33, 34]. The width of the fiber interval and the size of the nodules are independent predictors of portal hypertension.

The reversal of cirrhosis has become a research hotspot in recent years. Increasing clinical evidence shows that effective etiological treatments can reverse liver fibrosis/cirrhosis [35–39]. Bedossa [40] believes that the fibrous tissue in liver tissue degrades. Then, liver cells replace the disappearing fibrosis, resulting in the liver lobular structure returning to normal in order to consider cirrhosis reversal. According to pathophysiological mechanisms, the probability of cirrhosis reversal is higher if the occurrence of cirrhosis is recent, if etiology is controlled, if patients are young, or if nodular cirrhosis and avascular thrombosis are large.

The main clinical problem with the reversal of cirrhosis is the lack of reliable methods for measuring long-term changes in liver fibrosis. The Ishak fibrosis stage and Laennec cirrhosis scoring system, although commonly used, struggle to accurately assess dynamic changes in liver pathology. P-I-R classification can reflect dynamic changes in liver pathology [41]. The quantitative analysis and dynamic monitoring of liver fibrosis are more suitable for evaluating pathological changes related to decompensated cirrhosis.

According to China's Guidelines for the Diagnosis and Treatment of Cirrhosis, clinical cirrhosis can be divided into four critical periods, namely, the compensatory, decompensated, and recompensated periods, and cirrhosis reversal. In these guidelines, the criteria for the reversal of fibrotic cirrhosis include (1) a decrease in Ishak fibrosis stage by ≥1 or (2) a P-I-R classification decline after treatment [41]. Previous studies on liver pathology related to hepatitis B cirrhosis focused more on patients with chronic hepatitis B and compensated cirrhosis. Results suggested that effective antiviral therapy can improve liver histology and even end cirrhosis in some patients.

However, patients with decompensated cirrhosis often suffer from, for example, thrombocytopenia, abnormal coagulation function, and ascites. This significantly increases the risk of percutaneous liver biopsies. Although a transjugular liver biopsy can reduce the abovementioned risks, the necessary conditions are limited, and it is not widely applied in clinics. We hope that there will be relevant pathological data to support the recompensation of decompensated hepatitis B cirrhosis.

### 4.4. Limitations of Antiviral Therapy for Treatment of Decompensated Hepatitis B Cirrhosis

Current clinical studies showed that continuing viral suppression can recompensate partially decompensated HBV cirrhosis, and this recompensation is limited to some patients. After HBV replication is controlled, patients with HBV-related cirrhosis still have a risk of HCC. It is unclear whether patients who develop HCC after HBV replication is controlled still have cirrhosis or whether HCC occurrence is independent of cirrhosis reversal.

In addition to antiviral therapy, cell transplantation, antihepatic fibrosis therapy, and immunomodulatory therapy are hot research topics in the treatment of decompensated cirrhosis [42, 43]. Stem cells were proposed as an alternative to hepatocytes for cell transplantation. They are very attractive to the scientific community because of their high availability, good cell quality, and the possibility of using them in autologous cell transplantation [43]. Antihepatic fibrosis treatment is also a focus of cirrhosis treatment. Hepatic stellate cells are the central link of liver fibrosis. Peroxisome proliferator-activated receptor agonists and farnesate X receptor antagonists can inhibit hepatic-stellate-cell activation through related signaling pathways, thereby delaying the progression of fibrosis. There are also studies showing that statins can reduce portal hypertension in cirrhosis and even reduce the incidence and mortality of decompensation and HCC [44].

## 5. Summary and Outlook

There are many articles on the treatment and prognostic evaluation of decompensated hepatitis B cirrhosis; however, most articles do not provide original data, and some test indicators are different, making it difficult to compare the status of recompensation in hepatitis B cirrhosis.

Effective antiviral therapy can improve the liver biochemical indices of patients with decompensated hepatitis B cirrhosis. About 30% to 70% of patients have significantly improved CTP scores, suggesting that decompensated hepatitis B cirrhosis can be recompensated. However, the mechanism underlying liver cirrhosis and its related complications are not clear. At present, there are few studies comprehensively evaluating the long-term treatment effects of hepatitis B liver-cirrhosis-related complications, and on the recompensation of decompensated hepatitis B cirrhosis. Therefore, the evaluation time, evaluation indicators, influencing factors, and long-term prognosis of recompensated patients are still unclear. There is no parameter describing all recompensation characteristics. To explore the pathogenesis of recompensation of decompensated hepatitis B cirrhosis, further cohort studies and pathological research are needed. The identification of high-risk populations who struggle to achieve cirrhosis recompensation at an early stage, and the exploration of effective treatment strategies is hotspots in the field of liver disease at home and abroad. In short, how to clinically evaluate and achieve the recompensation of decompensated hepatitis B cirrhosis is still a contentious topic.

## Figures and Tables

**Table 1:** Oral antiviral therapy in HBV-related decompensated cirrhosis.

Year, (ref.)	Country	Drugs used	Number of patients	Follow-up period	ALT normalization (%)	HBV DNA undetectable (%)	MELD score	CTP score	HCC (%)	Liver transplantation (%)	Death (%)
2018, Jang JW	Korea	ETV/LAM	179/116	10 years	n.r.	<20 IU/ml 39.3%	Change from baseline LAM –3.28 ETV –5.00	Change from baseline LAM –3.09 ETV –2.40	20.0%	0-0.5y 10.5% 0.5-10 y	0-0.5y 6.8% 0.5-10y 25.8%
2017, Wan YM	China	LdT/LAM/ETV	31/45/54	2 years	LdT 83.3% LAM 64.3% ETV 85.4%	<500 copies/ml LdT 83.7% LAM 65.3% ETV 89.1%	Significantly improved compared to baseline.	Significantly improved compared to baseline.	LdT 15.0% LAM 15.6% ETV 14.8%	n.r.	LdT 20.0% LAM 6.7% ETV 12.7%
2017, Lee SK	Korea	TDF	57	1 year	77.2%	<116 copies/ml 70.2%	Change from baseline –2.9	Change from baseline –1.7	n.r.	n.r.	0%
2012, Chan HL	Global	LdT/LAM	116/116	2 years	LdT 61.4% LAM 52.4%	<300 copies/ml LdT 49.1% LAM 39.5%	n.r.	Decrease ≥ 2 point LdT 38.6% LAM 40.4%	LdT 15% LAM 16%	LdT 4.35% LAM 3.48%	LdT 16% LAM 22%
2011, Liaw YF	Taiwan	ETV (1 mg)/ADV	100/91	1 year	ETV 63% ADV 46%	<300 copies/ml ETV 57% ADV 20%	Change from baseline ETV –2.6 ADV –1.7	Improvement ETV 38% ADV 36%	ETV 12% ADV 20%	ETV 11% ADV 3%	ETV 23% ADV 33%
2011, Liaw YF	Global	TDF/FTC+TDF/ETV	45/45/22	1 year	TDF 56.8% FTC+TDF 75.6% ETV 54.5%	<400 copies/ml TDF 70.5% FTC+TDF 87.8% ETV 72.7%	Change from baseline TDF –2.0 FTC+TDF –2.0 ETV –2.0	Decrease ≥ 2 point TDF 25.9% FTC+TDF 48.0% ETV 41.7%	n.r.	5.6%	TDF 4.4% FTC+TDF 4.4% ETV 9.1%
2010, Shim JH	Korea	ETV	70	1 year	76.4%	<51 copies/ml 89.1%	Change from baseline –2.3	Change from baseline –1.5	6.9%	4.3%	12.9%
2007, Schiff E	Global	ADV	176	1 year	77%	<1000 copies/ml 59%	Change from baseline –2.0	n.r.	n.r.	n.r.	14.0%
2003, Hann HW	USA	LAM	75	1 year	55%	<0.7 MEq/ml 69%	n.r.	Decrease ≥ 2 point 31%	n.r.	n.r.	13.3%

ALT: alanine aminotransferase; HBV: hepatitis B virus; MELD: model for end-stage liver disease; CTP: Child–Turcotte–Pugh; HCC: hepatocellular carcinoma; ETV: entecavir; LAM: lamivudine; n.r.: not reported; LdT: telbivudine; ADV: adefovir; TDF: tenofovir disoproxil fumarate; FTC: emtricitabine.

## Data Availability

Not applicable.

## References

[1] (2015). Guidelines for the Prevention, Care and Treatment of Persons with Chronic Hepatitis B Infection. *Geneva*.

[2] Fattovich G., Bortolotti F., Donato F. (2008). Natural history of chronic hepatitis B: special emphasis on disease progression and prognostic factors. *Journal of Hepatology*.

[3] Samonakis D. N., Koulentaki M., Coucoutsi C. (2014). Clinical outcomes of compensated and decompensated cirrhosis: a long term study. *World Journal of Hepatology*.

[4] European Association for the Study of the Liver (2018). EASL Clinical Practice Guidelines for the management of patients with decompensated cirrhosis. *Journal of Hepatology*.

[5] Xu Y., Zhang Y. G., Wang X. (2015). Long-term antiviral efficacy of entecavir and liver histology improvement in Chinese patients with hepatitis B virus-related cirrhosis. *World Journal of Gastroenterology*.

[6] Lok A. S. (2013). Hepatitis: long-term therapy of chronic hepatitis B reverses cirrhosis. *Nature Reviews. Gastroenterology & Hepatology*.

[7] Young K., Liu B., Bhuket T. (2017). Long-term trends in chronic hepatitis B virus infection associated liver transplantation outcomes in the United States. *Journal of Viral Hepatitis*.

[8] Yang X., Li J., Zhou L., Liu J., Wang J., Lu W. (2014). Comparison of telbivudine efficacy in treatment-naive patients with hepatitis B virus-related compensated and decompensated cirrhosis in 96 weeks. *European Journal of Gastroenterology & Hepatology*.

[9] al-Hamoudi W., Elsiesy H., Bendahmash A. (2015). Liver transplantation for hepatitis B virus: decreasing indication and changing trends. *World Journal of Gastroenterology*.

[10] D’Amico G., Morabito A., D’Amico M. (2018). New concepts on the clinical course and stratification of compensated and decompensated cirrhosis. *Hepatology International*.

[11] D'Amico G., Pasta L., Morabito A. (2014). Competing risks and prognostic stages of cirrhosis: a 25-year inception cohort study of 494 patients. *Alimentary Pharmacology & Therapeutics*.

[12] Peng C. Y., Chien R. N., Liaw Y. F. (2012). Hepatitis B virus-related decompensated liver cirrhosis: benefits of antiviral therapy. *Journal of Hepatology*.

[13] Shim J. H., Lee H. C., Kim K. M. (2010). Efficacy of entecavir in treatment-naïve patients with hepatitis B virus-related decompensated cirrhosis. *Journal of Hepatology*.

[14] Liaw Y.-F., Raptopoulou-Gigi M., Cheinquer H. (2011). Efficacy and safety of entecavir versus adefovir in chronic hepatitis B patients with hepatic decompensation: a randomized, open-label study. *Hepatology*.

[15] Singal A. K., Fontana R. J. (2012). Meta-analysis: oral anti-viral agents in adults with decompensated hepatitis B virus cirrhosis. *Alimentary Pharmacology & Therapeutics*.

[16] Srivastava M., Rungta S., Dixit V. K., Shukla S. K., Singh T. B., Jain A. K. (2013). Predictors of survival in hepatitis B virus related decompensated cirrhosis on tenofovir therapy: an Indian perspective. *Antiviral Research*.

[17] Yue-Meng W., Li Y. H., Wu H. M. (2017). Telbivudine versus lamivudine and entecavir for treatment-naïve decompensated hepatitis B virus-related cirrhosis. *Clinical and Experimental Medicine*.

[18] Jang J. W., Choi J. Y., Kim Y. S. (2018). Effects of virologic response to treatment on short- and long-term outcomes of patients with chronic hepatitis B virus infection and decompensated cirrhosis. *Clinical gastroenterology and hepatology : the official clinical practice journal of the American Gastroenterological Association*.

[19] Lee S. K., Song M. J., Kim S. H. (2017). Safety and efficacy of tenofovir in chronic hepatitis B-related decompensated cirrhosis. *World Journal of Gastroenterology*.

[20] Chan H. L., Chen Y. C., Gane E. J. (2012). Randomized clinical trial: efficacy and safety of telbivudine and lamivudine in treatment-naïve patients with HBV-related decompensated cirrhosis. *Journal of Viral Hepatitis*.

[21] Liaw Y.-F., Sheen I.-S., Lee C.-M. (2011). Tenofovir disoproxil fumarate (TDF), emtricitabine/TDF, and entecavir in patients with decompensated chronic hepatitis B liver disease. *Hepatology*.

[22] Schiff E., Lai C. L., Hadziyannis S. (2007). Adefovir dipivoxil for wait-listed and post-liver transplantation patients with lamivudine-resistant hepatitis B: final long-term results. *Liver Transplantation*.

[23] Hann H. W., Fontana R. J., Wright T. (2003). A United States compassionate use study of lamivudine treatment in nontransplantation candidates with decompensated hepatitis B virus-related cirrhosis. *Liver Transplantation*.

[24] Manolakopoulos S., Triantos C., Theodoropoulos J. (2009). Antiviral therapy reduces portal pressure in patients with cirrhosis due to HBeAg-negative chronic hepatitis B and significant portal hypertension. *Journal of Hepatology*.

[25] Nagaoki Y., Aikata H., Daijyo K. (2018). Risk factors for exacerbation of gastroesophageal varices and portosystemic encephalopathy during treatment with nucleos(t)ide analogs for hepatitis B virus-related cirrhosis. *Hepatology Research : The Official Journal of the Japan Society of Hepatology*.

[26] Arvaniti V., D'Amico G., Fede G. (2010). Infections in patients with cirrhosis increase mortality four-fold and should be used in determining prognosis. *Gastroenterology*.

[27] Fede G., D’Amico G., Arvaniti V. (2012). Renal failure and cirrhosis: a systematic review of mortality and prognosis. *Journal of Hepatology*.

[28] European Association for the Study of the Liver (2019). EASL Clinical Practice Guidelines on nutrition in chronic liver disease. *Journal of Hepatology*.

[29] Bojko M. (2019). Causes of sarcopenia in liver cirrhosis. *Clinical Liver Disease*.

[30] Lim S. G., Aung M. O., Mak B. (2011). Clinical outcomes of lamivudine-adefovir therapy in chronic hepatitis B cirrhosis. *Journal of Clinical Gastroenterology*.

[31] Hyun J. J., Seo Y. S., Yoon E. (2012). Comparison of the efficacies of lamivudine versus entecavir in patients with hepatitis B virus-related decompensated cirrhosis. *Liver International*.

[32] Fontana R. J., Hann H. W., Perrillo R. P. (2002). Determinants of early mortality in patients with decompensated chronic hepatitis B treated with antiviral therapy. *Gastroenterology*.

[33] Deniz K., Özcan S., Özbakır Ö., Patıroğlu T. E. (2015). Regression of steatohepatitis-related cirrhosis. *Seminars in Liver Disease*.

[34] Bedossa P., Poynard T. (1996). An algorithm for the grading of activity in chronic hepatitis C. *Hepatology*.

[35] Chang T. T., Liaw Y. F., Wu S. S. (2010). Long-term entecavir therapy results in the reversal of fibrosis/cirrhosis and continued histological improvement in patients with chronic hepatitis B. *Hepatology*.

[36] Marcellin P., Asselah T. (2013). Long-term therapy for chronic hepatitis B: hepatitis B virus DNA suppression leading to cirrhosis reversal. *Journal of Gastroenterology and Hepatology*.

[37] Schiff E. R., Lee S. S., Chao Y. C. (2011). Long-term treatment with entecavir induces reversal of advanced fibrosis or cirrhosis in patients with chronic hepatitis B. *Clinical Gastroenterology and Hepatology*.

[38] Liaw Y. F. (2013). Reversal of cirrhosis: an achievable goal of hepatitis B antiviral therapy. *Journal of Hepatology*.

[39] Jung Y. K., Yim H. J. (2017). Reversal of liver cirrhosis: current evidence and expectations. *The Korean Journal of Internal Medicine*.

[40] Bedossa P. (2015). Reversibility of hepatitis B virus cirrhosis after therapy: who and why. *Liver international : official journal of the International Association for the Study of the Liver*.

[41] Sun Y., Zhou J., Wang L. (2017). New classification of liver biopsy assessment for fibrosis in chronic hepatitis B patients before and after treatment. *Hepatology*.

[42] Muthiah M. D., Huang D. Q., Zhou L. (2019). A murine model demonstrating reversal of structural and functional correlates of cirrhosis with progenitor cell transplantation. *Scientific reports*.

[43] Iansante V., Chandrashekran A., Dhawan A. (2018). Cell-based liver therapies: past, present and future. *Philosophical transactions of the Royal Society of London. Series B, Biological sciences*.

[44] Kamal S., Khan M. A., Seth A. (2017). Beneficial effects of statins on the rates of hepatic fibrosis, hepatic decompensation, and mortality in chronic liver disease: a systematic review and meta-analysis. *The American Journal of Gastroenterology*.

